# Diagnostic, clinicopathologic, therapeutic and prognostic value of Plasma Heat Shock Protein 90 levels in patients with advanced Gastrointestinal Carcinoma

**DOI:** 10.7150/jca.46343

**Published:** 2020-08-08

**Authors:** Yiyin Zhang, Longgang Ni, Qianqian Li, Min Li, Jiejie Zhu, Fei Zhang, Kangsheng Gu

**Affiliations:** 1 Department of Oncology, the First Affiliated Hospital of Anhui Medical University, Hefei, Anhui, 230022, P. R. China.; 2 Department of Oncology, Dongfang Cancer Hospital, Huainan, Anhui, 232000, P. R. China.; 3 Department of Medical Psychology, the First Affiliated Hospital of Anhui Medical University, Hefei, Anhui, 230022, P. R. China.

**Keywords:** Gastrointestinal carcinoma, HSP90, Peripheral blood

## Abstract

**Purpose:** Heat shock protein 90 (HSP90) is a critical molecular chaperone for protein folding, intracellular disposition and regulation of tumor biological behavior in the extracellular space. HSP90 has received much attention due to its specific effect in gastrointestinal cancer. This clinical study sought to determine whether HSP90 in plasma may serve as a biomarker in patients with advanced gastrointestinal carcinoma.

**Methods:** Using human plasma samples of advanced gastrointestinal carcinoma, we investigated the specific value of HSP90 in gastrointestinal cancer from a clinical perspective.

**Results:** In summary, plasma levels of HSP90 were shown to be higher in patients with gastric cancer (GC) or colorectal cancer (CRC) than in controls with benign gastrointestinal diseases. In both GC and CRC patients, HSP90 was significantly associated with live metastasis. Higher HSP90 levels were more frequent in CRC patients with hazardous or harmful alcohol consumption habits. Patients with RAS mutations had higher HSP90 levels in CRC. Compared with Carcinoembryonic Antigen (CEA) and Carbohydrate Antigen 19-9 (CA19-9), HSP90 benefited patients by enhancing diagnostic sensitivity and the Youden index. The levels of HSP90 were inversely associated with short-term efficacy in GC patients who had received fluorouracil/platinum-based advanced first-line treatment. When first-line therapy failed, plasma HSP90 levels in patients with GC were significantly increased. In terms of progression-free survival (PFS), patients with GC or CRC who had low levels of HSP90 were not significantly different from those with high levels of HSP90. Univariate and multivariate analyses demonstrated that HSP90 was not an independent prognostic predictor for GC and CRC patients with PFS. However, RAS mutation was an independent prognostic factor for poor PFS in CRC patients.

**Conclusions:** Plasma HSP90 levels have potential diagnostic value in advanced gastrointestinal carcinoma and therapeutic predictive value in GC.

## Introduction

Gastrointestinal carcinoma is one of the most familiar types of carcinoma and one of the leading causes of cancer-related death worldwide 1,2. Conventional medical treatment of advanced gastrointestinal carcinoma involves chemotherapy, targeted therapy and immunotherapy. Nevertheless, standard therapies provide only a limited survival benefit.

At present, the peripheral blood levels of Carbohydrate Antigen 19-9 (CA19-9) and Carcinoembryonic Antigen (CEA) are commonly used to guide the management of gastrointestinal carcinoma. However, these biomarkers exhibit poor sensitivity and specificity in some patients. Therefore, the identification of novel biomarkers is important for monitoring the response to therapy and predicting the effect in patients with advanced gastrointestinal carcinoma.

Heat shock proteins (HSPs) constitute a series of proteins induced by heat shock or cellular stresses that are able to prevent apoptosis and misfolding of proteins in the cell. According to their molecular weights, HSPs are divided into small HSPs, Hsp60, Hsp70 and Hsp90. In particular, it has been showed that HSPs are significantly associated with many kinds of cancers 3. Among these proteins, Heat Shock Protein 90 (HSP90) is a critical molecular chaperone for protein folding, intracellular disposition and regulation of tumor biological behaviour in the extracellular space 4,5. At present, HSP90 has received much attention due to its specific effect in certain types of cancer, especially in gastrointestinal cancer. It has been shown that high expression of HSP90 in tumor tissue is associated with tumor aggressiveness and poor prognosis in patients with advanced gastric cancer (GC) 6. Upregulation of Hsp90 in tumor tissue correlates with metastasis in GC 7. In contrast, immunohistochemical analyses of HSP90 and HER-2 expression revealed better prognostic relevance in GC tissue in another investigation 8. Similar studies have been performed on colorectal cancer (CRC). For example, HSP90 in plasma confers an advantage in the diagnosis of early CRC 9. However, whether HSP90 can be used as a biomarker for the evaluation of therapeutic effects is unknown. Moreover, the prognostic value of HSP90 expression in gastrointestinal cancer patients is also controversial and incompletely evaluated.

In particular, the analyses of HSP90 expression are mainly based on tumor tissue from an operative resection or endoscope. Thus, repeated detection of HSP90 before and after treatment using tumor tissue is complicated. Invasive procedures cause patients to experience more pain. For these reasons, clinical practices require a simple and more convenient method of HSP90 detection through peripheral blood from gastrointestinal cancer patients.

Therefore, this clinical study sought to determine whether HSP90 may serve as a biomarker in patients with advanced gastrointestinal carcinoma. Specifically, our aims were as follows: i) to assess the correlation between levels of HSP90 and clinicopathologic features; ii) to examine the diagnostic value between HSP90 and current biomarkers, such as CEA and CA19-9; iii) to determine the change in HSP90 before and after first-line treatment and the relationship between HSP90 and short-term efficacy; iv) to show the correlation between HSP90 and the progression-free survival (PFS) of patients with advanced gastrointestinal carcinoma who have received first-line treatment; and v) to evaluate the correlation between the level of HSP90 and prognosis.

## Materials and Methods

### Patients and treatment

The study was approved by the Ethics Committee of Anhui Medical University. We analyzed peripheral blood plasma from 186 patients with advanced gastrointestinal adenocarcinoma (103 gastric and 83 colorectum) who were treated at the First Affiliated Hospital of Anhui Medical University from March 2018 to March 2020. Inclusion criteria were as follows: i) each subject signed an informed consent form before entry into this study; ii) all patients were diagnosed with gastric or colorectal adenocarcinoma through histopathological examination; iii) all patients were initially diagnosed before any treatment; iv) all patients had advanced metastatic and unresectable cancer; v) the physical condition and main organ function of all patients were amenable to targeted therapy or chemotherapy; vi) the clinicopathological data were relatively complete. The exclusion criteria were as follows: i) the patient cases were associated with acute or uncontrolled infectious disease; ii) the patient cases were associated with severe cardiovascular and cerebrovascular diseases or rheumatism; and iii) the patients were diagnosed with multiple primary malignancies or other pathological types. Pre- or posttherapeutic plasma samples were collected. The staging of these patients with gastrointestinal adenocarcinoma was classified according to the American Joint Committee on Cancer classification (AJCC, 8th edition). Clinicopathologic data, including age, sex, differentiation, level of CEA and CA19-9, liver metastasis, lymph node metastasis, lung metastasis, peritoneal metastasis, alcohol consumption, and molecular classification, were evaluated by reviewing the patients' medical records. On the other hand, plasma samples were collected from patients with gastrointestinal (50 gastric cases and 46 colorectal cases) benign disease as controls. The exclusion criteria of the control group were consistent with those of the experimental group.

Advanced first-line treatment (chemotherapy or targeted therapy combined with chemotherapy) was administered at the discretion of the physician and agreement of the patients until the disease progressed. Short-term efficacy was evaluated after two treatment cycles according to the standard Response Evaluation Criteria in Solid Tumors (RECIST, 1.1 edition) as four outcome measures, including complete response (CR), partial response (PR), stable disease (SD), and progressive disease (PD). CR+PR was used to calculate the objective response rate (ORR), and CR+PR+SD was used to describe the disease control rate (DCR). Moreover, analyses for PFS were also performed. PFS was calculated from the time of diagnosis to disease progression or the last follow-up evaluation. In this study, the follow-up period ended on March 1, 2020.

### Blood collection and enzyme-linked immunosorbent assay

Peripheral blood samples were collected from all patients with advanced gastrointestinal adenocarcinoma prior to medical treatment and when evaluating the efficacy of the treatment. Plasma was separated from whole blood through centrifugation. Plasma HSP90 was measured with a commercially available ELISA kit (Protgen, China) according to the manufacturer's recommendations.

Diluted plasma samples and standard samples were added to a 96-well microplate precoated with HSP90 antibody. HRP-conjugated anti-HSP90 antibody was added to the plate and the plate was immediately incubated at 37 °C for 1 hour. The reaction was visualized by adding chromogen 3,3,5,5-tetramethylbenzidine (TMB) solutions A and B to each well sequentially and incubated at 37 degrees centigrade. Importantly, the reaction was terminated by adding stop solution to each well. The optical density was measured at 450 nm on a spectrophotometer (BioTek Synergy HTX, USA). The standard curve was produced and the amount of HSP90 was determined in the plasma sample.

### Statistical analysis

Statistical analysis was performed using SPSS 17.0 edition (SPSS, USA) and GraphPad Prism software 6.0 edition (GraphPad, USA). Student's t-test was used to evaluate the associations between HSP90 levels and clinicopathologic factors. The correlation between the blood tumor biomarkers was assessed by the Pearson correlation coefficient. The association between cancer and benign disease control HSP90 expression was tested using Student's t-test. The diagnostic performance of HSP90 and other biomarkers was evaluated using receiver operating characteristic (ROC) curves. Paired Student's t-test was performed to assess differences in HSP90 before and after treatment. The Kaplan-Meier method and the log-rank test were used to estimate the survival distributions. Univariate and multivariate analyses were performed by Cox proportional hazards regression modeling. The significance level was set at less than 0.05.

## Results

### Clinicopathologic and clinical features of patients

**Figure 1:**
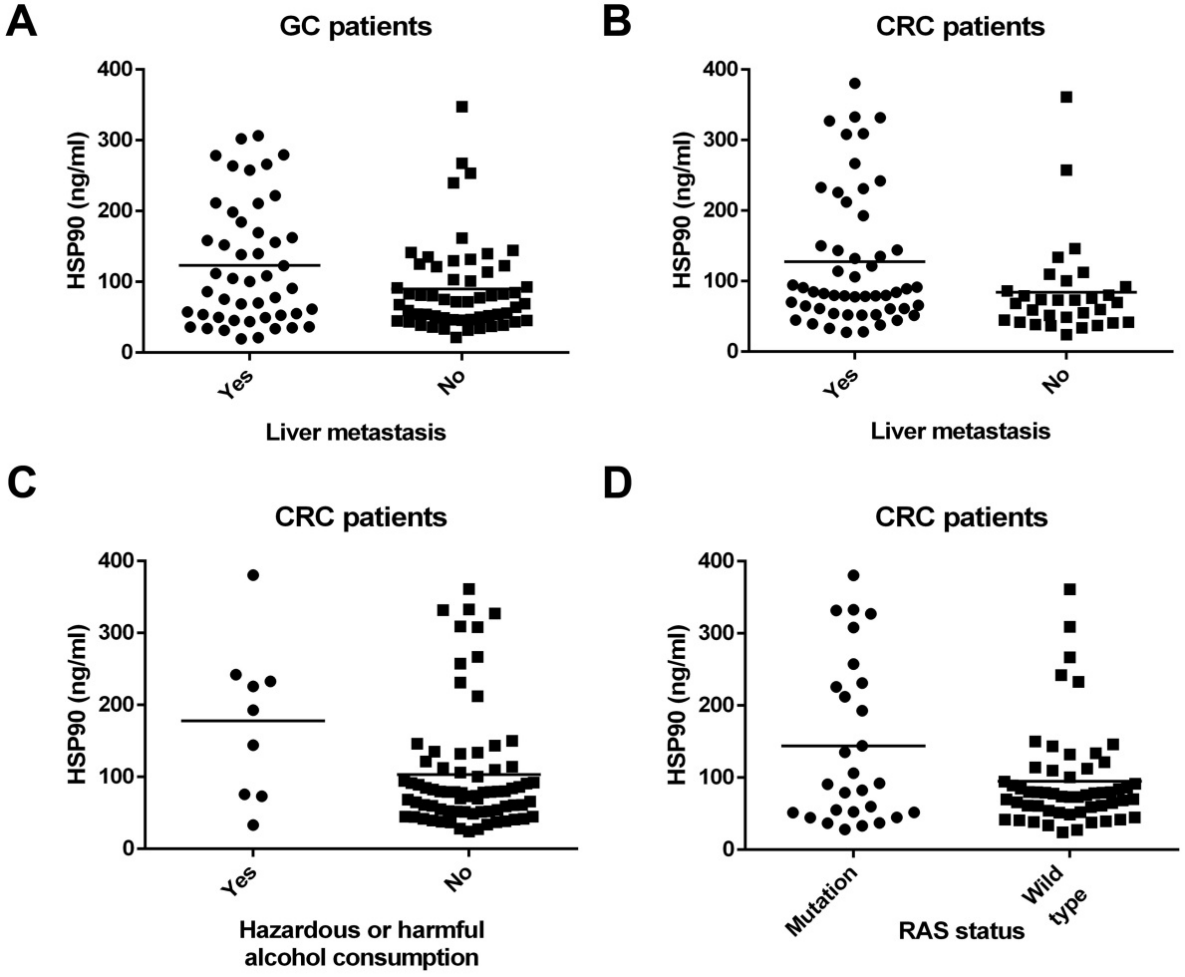
** Correlations between clinicopathologic features and levels of HSP90 in patients with GC and CRC.** HSP90 was significantly associated with liver metastasis in patients with GC (**A**) and CRC (**B**). Higher HSP90 levels were more frequent in CRC patients with hazardous or harmful alcohol consumption habits (**C**). Patients with RAS mutations had higher HSP90 levels in CRC (**D**).

A total of 186 subjects were enrolled in this study, which comprised 103 pathologically proven GCs and 83 CRCs. The characteristics of all the patients are presented in Table 1. Tumor differentiation was not obtained from 18 patients with GC and CRC. In both GC (P=0.030) (Figure 1A) and CRC (P=0.019) (Figure 1B) patients, HSP90 was significantly associated with liver metastasis (Table 1). Patients with liver metastasis had higher HSP90 levels in plasma. However, there were no relationships between the HSP90 levels from all the patients and clinicopathologic factors, including age, sex, degree of differentiation, tumor location, lymph node metastasis, lung metastasis, and peritoneal metastasis. In particular, higher HSP90 levels were more frequent in CRC patients with hazardous or harmful alcohol consumption habits (P=0.016) (Table 1) (Figure 1C). However, this trend was not observed in GC patients. In terms of molecular classification, patients with RAS mutations had higher HSP90 levels in CRC (P=0.042) (Figure 1D). Nevertheless, there were no relationships between HSP90 levels and the state of HER2/BRAF in GC or CRC patients. In the control group, there were no correlations between HSP90 levels and clinical characteristics (Table 2).

Five patients with GC and 2 patients with left hemicolon or rectal cancer did not receive advanced first-line therapy. The specific treatment plan is shown in Table 3. Short-term efficacy was evaluated in 81 GC patients and 57 CRC patients. PFS was recorded for 60 GC patients and 47 CRC patients. For these GC patients, the median follow-up period was 9.2 months (range, 1.6 to 21.2 months). Furthermore, the time range for follow-up evaluation in CRC patients was 0.8 to 23.1 months, with a median time of 10.3 months.

### Diagnostic value of HSP90, CEA and CA19-9

**Figure 2:**
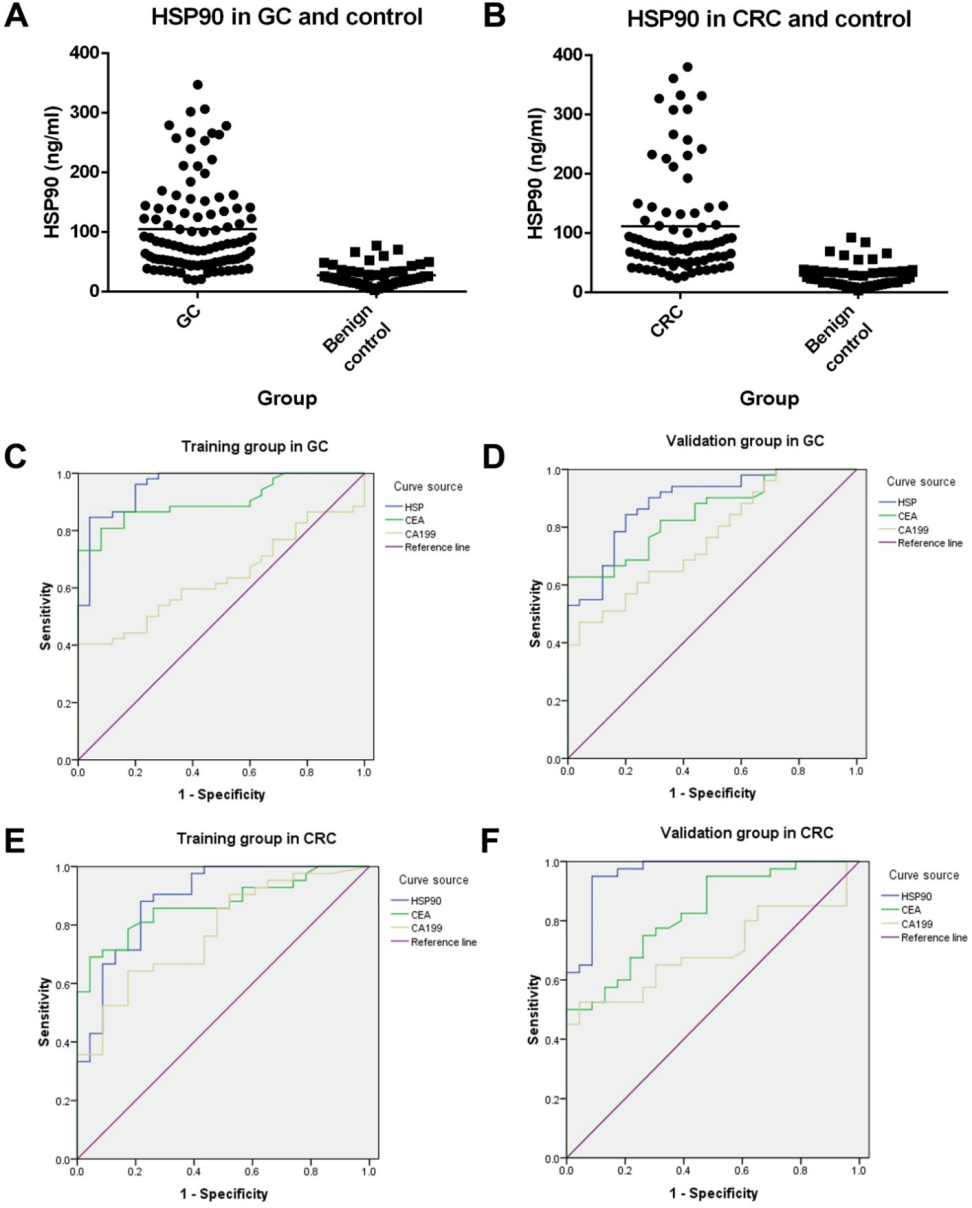
** Diagnostic value of HSP90, CEA and CA19-9 in patients with GC and CRC.** Plasma levels of HSP90 were analyzed by ELISA in advanced GC (**A**) or CRC (**B**) patients and controls. ROC curves of HSP90, CEA and CA19-9 for GC in the training group (**C**) and validation group (**D**). ROC curves of HSP90, CEA and CA19-9 for CRC in the training group (**E**) and validation group (**F**).

There was an extremely weak correlation between HSP90 and CEA in advanced GC patients (r=0.214, P=0.030). However, there was no correlation between HSP90 and CA19-9 in GC patients. Moreover, there was no association with HSP and CEA/CA19-9 in CRC patients. Plasma levels of HSP90 were shown to be higher in patients with GC (105.00 ng/ml vs 27.37 ng/ml, P<0.001) or CRC (111.40 ng/ml vs 28.60 ng/ml, P<0.001) than in gastrointestinal benign disease controls (Figure 2A and 2B).

To evaluate the diagnostic value of HSP90 in GC, the levels of HSP90 were compared with CEA and CA19-9 through ROC curves. In total, 103 GC patients and 50 gastric benign disease patients were collected. All these patients were divided into two groups randomly, containing 77 cases (52 GC patients and 25 controls) as the training group and 76 cases (51 GC patients and 25 controls) as the validation group. There were no significant relationships between the clinicopathological characteristics of patients with gastric diseases in the training group and validation group (Table 4). In the training cohort, the AUCs of HSP90, CEA and CA19-9 were 0.956, 0.903 and 0.636, respectively. Moreover, the AUCs of HSP90, CEA and CA19-9 were 0.888, 0.847 and 0.762, respectively, in the validation cohort. On the other hand, 83 CRC patients and 46 colorectal benign disease patients were enrolled. Sixty-five cases (42 CRC patients and 23 controls) were used as the training group, and 64 cases (41 CRC patients and 23 controls) were used as the validation group. There were no significant correlations between the clinicopathological characteristics of patients with colorectal diseases in the training group and validation group (Table 4). In the training cohort, the AUCs of HSP90, CEA and CA19-9 were 0.888, 0.869 and 0.777 in CRC patients, respectively. The AUCs of HSP90, CEA and CA19-9 were 0.962, 0.828 and 0.699 in the validation cohort, respectively. Thus, HSP90 had a better diagnostic performance than CEA or CA19-9 for GC and CRC through the Youden index (Table 5).

### Relationships between the level of HSP90 and short-term efficacy

**Figure 3:**
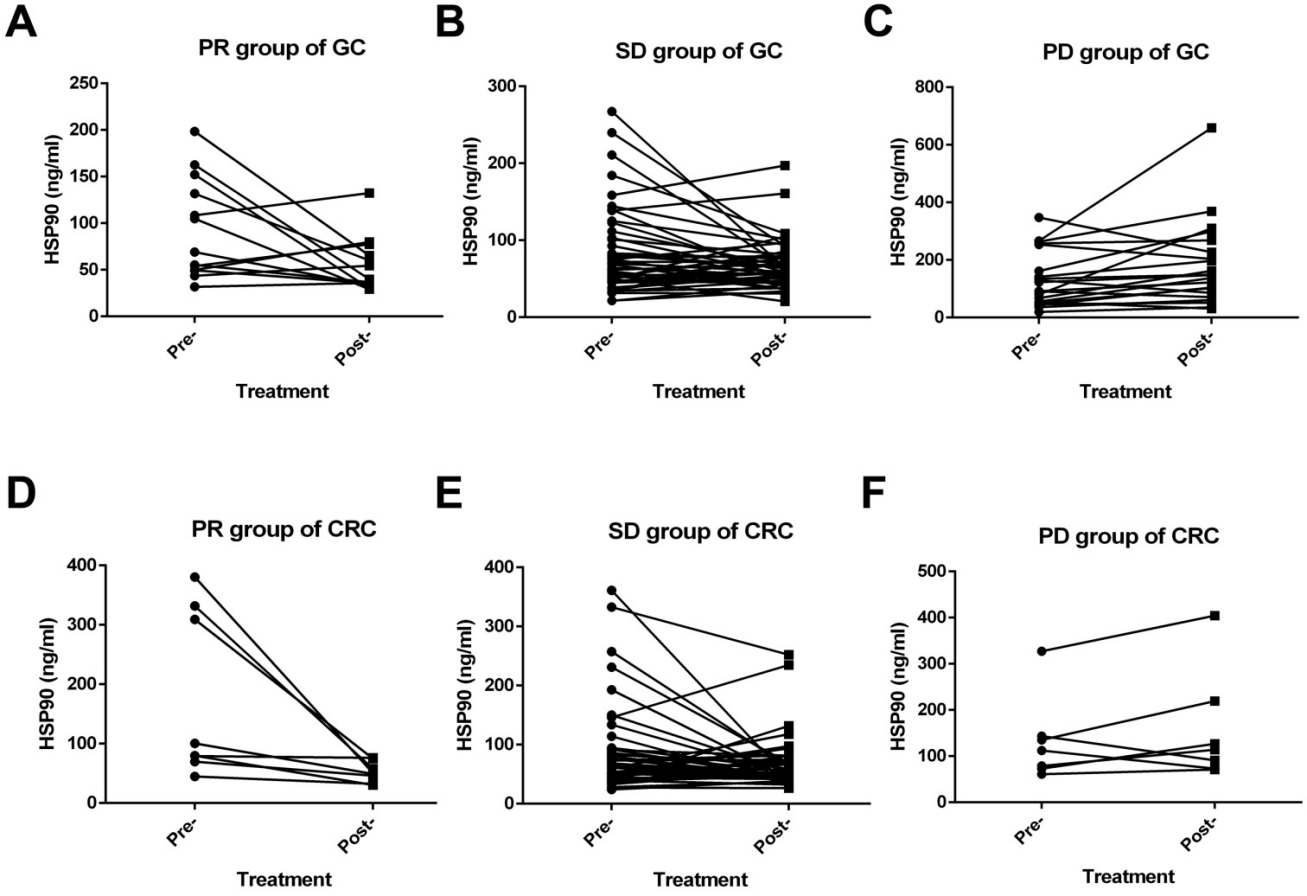
** Efficacy of advanced first-line treatment according to the levels of HSP90 in patients with GC and CRC.** A significant decrease in HSP90 levels was noticed with PR (**A**) and SD (**B**) outcomes after first-line therapy in GC. A significant increase in HSP90 levels was observed with PD (**C**) outcomes after first-line therapy in GC. In patients with CRC, the levels of HSP90 before first-line treatment were significantly higher than those after two cycles of treatment in the PR (**D**) group. HSP90 levels in SD (**E**) and PD (**F**) cases were not significantly changed before and after first-line therapy.

Among the GC patients, the short-term efficacy of first-line treatment was evaluated in 81 cases. These patients had received at least one fluorouracil or platinum regimen. None achieved CR, 13 patients achieved PR, 21 patients had PD, and 47 patients were in stable condition. Moreover, the ORR and DCR were 16.0% and 74.1%, respectively. A significant decrease in HSP90 levels was noticed with PR (P=0.039) (Figure 3A) and SD (P=0.042) (Figure 3B) outcomes after first-line therapy. Conversely, a significant increase in the levels of HSP90 was noticed with PD (p=0.040) (Figure 3C) outcomes after first-line therapy. On the other hand, 57 CRC patients could evaluate the short-term efficacy of first-line treatment. CR, PR, SD and PD were observed in 0, 8, 42, and 7 CRC patients, respectively. The ORR and DCR were 14.0% and 87.7%, respectively. In the PR (P=0.036) (Figure 3D) group, similar to the GC group, the levels of HSP90 before first-line treatment were significantly higher than those after two cycles of treatment. However, HSP90 expression in SD (P=0.063) (Figure 3E) and PD (P=0.283) (Figure 3F) cases was not significantly changed before and after first-line therapy.

### PFS of first-line therapy according to the level of HSP90

**Figure 4:**
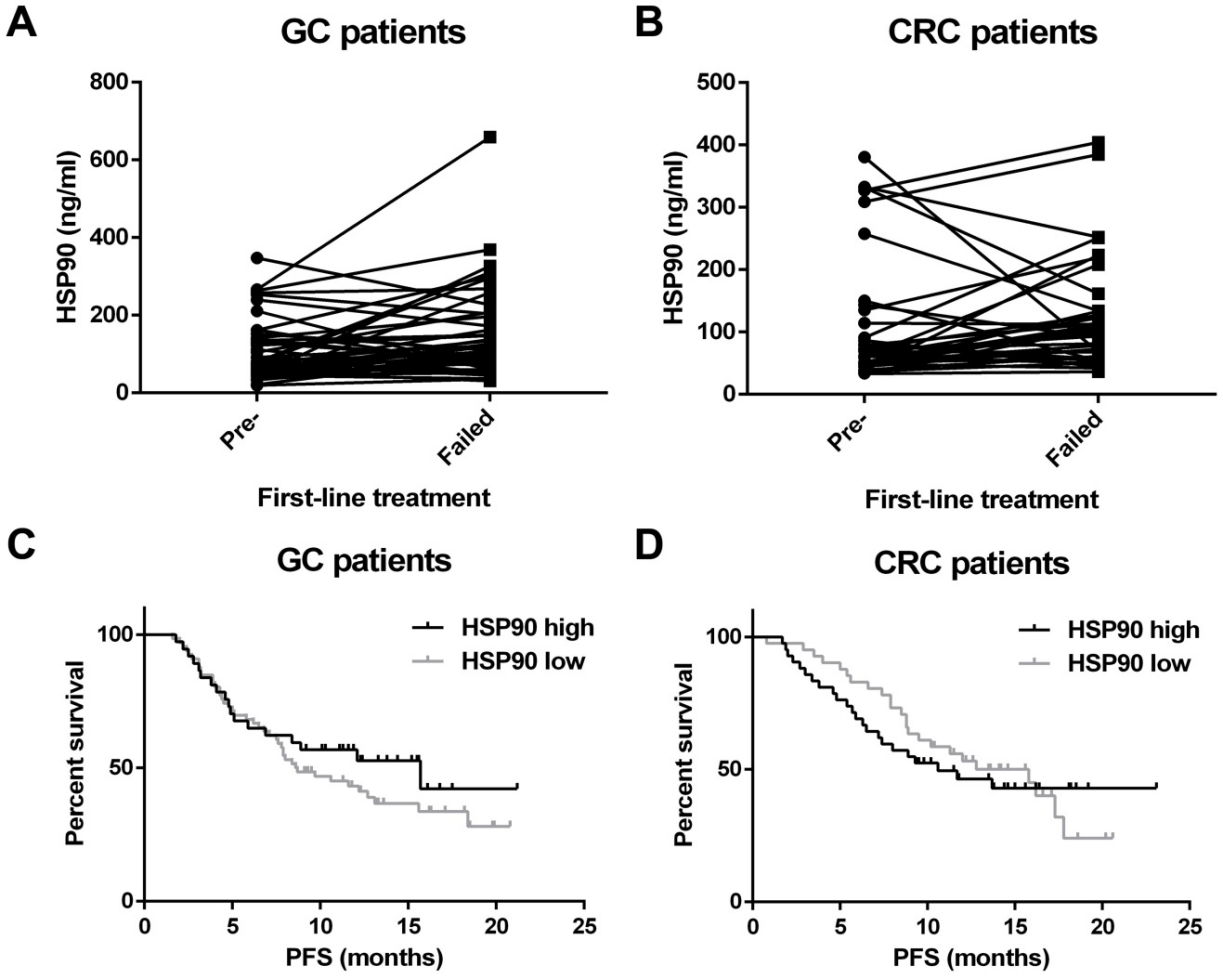
** Survival analysis for HSP90.** Plasma HSP90 levels in patients with GC (**A**) were significantly increased when first-line therapy failed. HSP90 levels in patients with CRC (**B**) were not significantly changed when first-line therapy failed. There was no correlation between the baseline levels of HSP90 and the PFS of patients with GC (**C**) and CRC (**D**) who had received advanced first-line treatment.

In total, the HSP90 level was recorded when initial diagnosis and treatment failed for 52 GC patients and 37 CRC patients. Interestingly, plasma HSP90 levels in patients with GC were significantly increased when first-line therapy failed (P=0.004) (Figure 4A). Unfortunately, a similar increase was not observed in patients with CRC (P=0.415) (Figure 4B).

All patients were divided into two groups based on the median HSP90 level in plasma. The patients with low levels of HSP90 were not significantly different from those with high levels of HSP90 in terms of PFS (GC: 8.7 versus 15.7 months, P=0.316; CRC: 12.8 versus 10.6 months, P=0.744) (Figure 4C and 4D).

### Prognostic value of HSP90 in patients with advanced GC and CRC

Univariate and multivariate analyses demonstrated that HSP90 was not an independent prognostic predictor for GC and CRC patients with PFS (Table 6). Furthermore, RAS mutation was an independent prognostic factor for poor PFS in CRC patients according to both the univariate analysis (HR: 2.138, 95% CI: 1.194-3.827, P=0.011) and the multivariate analysis (HR: 2.587, 95% CI: 1.273-5.260, P=0.009).

## Discussion

It has been reported that peripheral blood biomarkers can reflect the biological features of gastrointestinal cancer 10. In particular, HSP90 is released into the extracellular space as “eHSP90”, where it is involved in the controllability of tumor progression and metastasis 5. In this study, we found that plasma levels of HSP90 were higher in patients with GC and CRC than in controls with benign gastrointestinal diseases. This suggests that overexpression of HSP90 may be involved in the occurrence and development of gastrointestinal carcinoma. Moreover, based on these results, HSP90 had a higher Youden index value and larger AUC than CEA and CA19-9, consistent with the diagnostic value for gastrointestinal carcinoma patients in the training cohort. Furthermore, the above results were verified by the validation cohort. It is well known that CEA and CA19-9 are the standard markers for gastrointestinal carcinoma. This discovery of HSP90 as a new biomarker could benefit patients through enhanced diagnostic sensitivity and accuracy.

Furthermore, HSP90 functions in metastatic pathways by maintaining the stability of a wide range of molecular targets, such as epidermal growth factor receptor (EGFR), vascular endothelial growth factor receptor (VEGFR) and the RAS/RAF pathway 11,12. The liver is the most common metastatic site of gastrointestinal carcinoma. Through the hepatic portal vein, tumor cells from the gastrointestinal tract enter the liver. Moreover, it is significant that levels of HSP90 increase abnormally in both primary and secondary hepatic carcinoma 13. In this study, we discovered that patients with GC and CRC with liver metastasis had higher HSP90 levels in plasma, which indicated that the level of HSP90 from peripheral blood is closely related to the liver tumor burden. On the other hand, we found that patients with RAS mutations had higher HSP90 levels in CRC. Notably, mutated RAS may predict sensitivity to HSP90 inhibition due to serine threonine kinase 33-dependent mechanisms in CRC cell lines 14. According to the tumor tiuuse of CRC, another clinical study has confirmed that a positive correlation exists between KRAS mutation and HSP90 expression 15. These studies echo the findings of our study. In addition, we clarified that higher HSP90 levels were more frequent in CRC patients with hazardous or harmful alcohol consumption habits. The mechanism is still unclear but is suspected to be related to alcohol-induced inflammation and stress.

In terms of the efficacy evaluation, it is worth noting that inhibition of HSP90 promotes the efficacy of anticancer drugs 16. Fluorouracil and platinum are the basic chemotherapeutic drugs used for GC 17. HSP70/HSP90-organizing protein may protect GC cells from fluorouracil chemotherapy in an autocrine manner 18. The HSP90 inhibitor synergizes with platinum and regulates the proliferation and invasion of GC cell lines 19. Increased HSP90 or lysine demethylase 5B can facilitate the recruitment of X-ray repair cross complement 1 to repair DNA damaged by platinum, and inhibition of HSP90 can reverse chemoresistance in GC 20. Meanwhile, an HSP90 inhibitor combined with cisplatin can markedly suppress the growth of xenograft tumors in GC 21. In our research, we discovered that the levels of HSP90 were inversely associated with short-term efficacy in GC. Specifically, the levels of HSP90 were decreased after therapy in GC patients who achieved objective remission or disease control. However, the levels of HSP90 increased significantly as the disease progressed. The results of clinical studies are consistent with those in cell lines and xenograft animal models. Unfortunately, HSP90 did not fully reflect the short-term efficacy of first-line treatment in patients with CRC although HSP90 inhibition downregulates thymidylate synthase and thus synergizes with fluoropyrimidine-based chemotherapy in CRC cell lines and xenograft models 22,23.

In this study, the baseline levels of HSP90 were not correlated with PFS or prognosis in either GC or CRC. This finding may be related to many factors affecting the survival and prognosis of patients. Moreover, the sample size and follow-up time need to be expanded and extended. Interestingly, clinical trials have shown that the outcome of patients with wild-type KRAS is significantly better than that of patients with KRAS mutations 24,25. Our study obtained similar results according to RAS status for poor PFS.

Thus, in the present investigation, the diagnostic value of HSP90 in gastrointestinal cancer was superior to that of traditional tumor markers in peripheral blood. The level of HSP90 in plasma was associated with the fluorouracil/platinum-based short-term therapeutic effect in GC. At the same time, HSP90 was detected in plasma instead of tumor tissue, which was less invasive and greatly facilitated repeated testing. Compared with previous studies, the above specific novel findings can provide more convenience to clinical practice and improve the individualized diagnosis and treatment of gastrointestinal cancer.

## Conclusions

In summary, plasma levels of HSP90 were shown to be higher in patients with GC or CRC than in controls with benign gastrointestinal disease. In both GC and CRC patients, HSP90 was significantly associated with live metastasis. Higher HSP90 levels were more frequent in CRC patients with hazardous or harmful alcohol consumption habits. In terms of molecular classification, patients with RAS mutations had higher HSP90 levels in CRC. As a plasma biomarker, HSP90 could benefit patients by enhancing diagnostic sensitivity and the Youden index. The levels of HSP90 were inversely associated with short-term efficacy in GC patients who had received fluorouracil/platinum-based advanced first-line treatment. However, RAS mutation but not HSP90 was an independent prognostic factor for poor PFS in CRC patients. Our results suggest that plasma HSP90 levels have potential diagnostic value in advanced gastrointestinal carcinoma and therapeutic predictive value in GC.

## Figures and Tables

**Table 1:** Characteristics of HSP90 in patients of GC and CRC

Characteristics	GC patients			CRC patients		
	Cases (N)	Average HSP90 value (ng/ml)	*P*	Cases (N)	Average HSP90 value (ng/ml)	*P*
**Age (years)**						
≥60	69	102.53	0.637	36	118.60	0.518
<60	34	110.13		47	105.81	
**Sex**						
Male	86	104.49	0.872	52	115.00	0.630
Female	17	107.79		31	105.25	
**Differentiation**						
Poor	46	96.00	0.364	12	134.74	0.213
Moderate and well	39	111.66		53	100.80	
**Live metastasis**						
Yes	47	123.27	0.030	52	127.58	0.019
No	56	89.73		31	84.15	
**Lymph node metastasis**						
Yes	75	111.76	0.145	56	108.67	0.693
No	28	87.03		27	116.93	
**Lung metastasis**						
Yes	23	122.28	0.221	35	101.95	0.412
No	80	100.08		48	118.22	
**Peritoneal metastasis**						
Yes	26	114.10	0.487	15	106.99	0.834
No	77	101.98		68	112.32	
**Alcohol consumption**						
Hazardous or harmful	19	98.59	0.686	9	177.85	0.016
Abstinence or low risk	84	106.50		74	103.27	
**Molecular classification**						
HER2 positive	10	104.14	0.969	-	-	-
HER2 negative	93	105.13		-	-	
RAS mutation	-	-	-	28	143.79	0.042
RAS wild type	-	-		55	94.84	
BRAF mutation	-	-	-	5	126.15	0.703
BRAF wild type	-	-		78	110.41	
**CRC location**						
Right hemicolon	-	-	-	23	125.22	0.381
Left hemicolon and rectum	-	-		60	106.04	

**Table 2:** Characteristics of HSP90 in patients with gastrointestinal benign disease as controls

Characteristics	Patients with gastric benign disease			Patients with colorectal benign disease		
	Cases (N)	Average HSP90 value (ng/ml)	*P*	Cases (N)	Average HSP90 value (ng/ml)	*P*
**Age (years)**						
≥60	31	30.27	0.132	22	29.90	0.684
<60	19	22.65		24	27.40	
**Sex**						
Male	29	26.49	0.676	23	29.94	0.660
Female	21	28.59		23	27.25	

**Table 3:** Advanced first-line treatment plan in patients of GC and CRC

Tumor location	Molecular classification	Treatment regime	Cases
Gastric	HER2 positive	Trastuzumab+Fluorouracil+Platinum	6
		Trastuzumab+Fluorouracil+Taxanes	3
		Others	1
	HER2 negative	Fluorouracil+Platinum	47
		Fluorouracil+Taxanes	23
		Others	18
Left hemicolon and rectum	RAS and BRAF wild type	Cetuximab+Irinotecan+Fluorouracil	2
		Cetuximab+Oxaliplatin+Fluorouracil	2
		Others	1
	RAS or BRAF mutation	Bevacizumab+Irinotecan+Fluorouracil	9
		Bevacizumab+Oxaliplatin+Fluorouracil	20
		Others	24
Right hemicolon	-	Bevacizumab+Irinotecan+Fluorouracil	9
	-	Bevacizumab+Oxaliplatin+Fluorouracil	8
		Others	6

**Table 4:** The correlations about clinicopathological characteristics of patients between training group and validation group

Characteristics	Gastric		Colorectum	
	Statistic	*P* value	Statistic	*P* value
Age	0.203	0.652	1.786	0.181
Sex	0.002	0.963	0.992	0.319
Differentiation	0.101	0.751	0.119	0.730
Liver metastasis	1.676	0.195	0.097	0.755
Lymph node metastasis	0.682	0.409	2.446	0.118
Lung metastasis	1.277	0.258	0.017	0.898
Peritoneal metastasis	0.723	0.395	0.055	0.815
Alcohol consumption	0.091	0.763	0.153	0.696
HER2 status	0.133	0.715	-	-
RAS status	-	-	0.149	0.699
BRAF status	-	-	<0.001	1.000
Tumor location	-	-	3.186	0.074

**Table 5:** Diagnostic performance of HSP90 in patients with GC and CRC

Biomarkers		Training group				Validation group			
		Sensitivity	Specificity	AUC	Youden index value	Sensitivity	Specificity	AUC	Youden index value
GC	HSP90	84.6%	96.0%	0.956	0.806	84.3%	80.0%	0.888	0.643
	CEA	73.1%	100.0%	0.903	0.731	62.7%	100.0%	0.847	0.627
	CA19-9	40.4%	100.0%	0.636	0.404	47.1%	96.0%	0.762	0.431
CRC	HSP90	88.1%	78.3%	0.888	0.664	95.0%	91.3%	0.962	0.863
	CEA	69.0%	95.7%	0.869	0.647	50.0%	100.0%	0.828	0.500
	CA19-9	64.3%	82.6%	0.777	0.469	52.5%	95.7%	0.699	0.482

**Table 6:** Univariate and multivariate analysis of factors according to PFS in advanced GC and CRC patients

Factors	GC						CRC					
	Univariate analysis			Multivariate analysis			Univariate analysis			Multivariate analysis		
	HR	95 % CI	*P* value	HR	95% CI	*P* value	HR	95% CI	*P* value	HR	95% CI	*P* value
HSP90	0.755	0.434-1.312	0.318	0.612	0.315-1.186	0.146	1.100	0.620-1.951	0.744	0.980	0.474-2.026	0.956
Sex	1.012	0.513-1.997	0.972	0.999	0.440-2.270	0.999	1.377	0.744-2.546	0.308	1.520	0.725-3.186	0.268
Age	0.626	0.374-1.047	0.074	0.763	0.419-1.389	0.376	0.965	0.536-1.738	0.906	0.557	0.241-1.285	0.170
Differentiation	1.277	0.730-2.236	0.392	1.156	0.622-2.149	0.647	1.303	0.564-3.010	0.536	2.040	0.774-5.375	0.149
Lung metastasis	1.026	0.553-1.904	0.934	1.328	0.652-2.705	0.435	0.884	0.493-1.585	0.679	1.459	0.649-3.278	0.361
Liver metastasis	1.411	0.847-2.349	0.186	1.489	0.801-2.767	0.208	1.195	0.653-2.188	0.563	1.286	0.602-2.746	0.516
Lymph node metastasis	1.212	0.666-2.206	0.529	1.138	0.574-2.258	0.711	0.823	0.450-1.507	0.529	0.657	0.286-1.511	0.323
Peritoneal metastasis	0.665	0.386-1.147	0.143	0.570	0.308-1.055	0.073	1.026	0.478-2.202	0.947	1.155	0.418-3.193	0.781
Alcohol consumption	0.774	0.392-1.529	0.461	0.693	0.298-1.613	0.395	0.986	0.389-2.500	0.976	1.403	0.468-4.205	0.546
HER2 status	0.838	0.381-1.845	0.661	0.930	0.369-2.343	0.878	-	-	-	-	-	-
RAS status	-	-	-	-	-	-	2.138	1.194-3.827	0.011	2.587	1.273-5.260	0.009
BRAF status	-	-	-	-	-	-	1.220	0.377-3.946	0.740	0.365	0.042-3.194	0.362
Tumor location	-	-	-	-	-	-	0.928	0.481-1.790	0.823	0.761	0.319-1.819	0.539
